# “Intraperitoneal ruptured hydatid cyst of liver with cystobiliary communication: A case report”

**DOI:** 10.1016/j.ijscr.2025.111012

**Published:** 2025-02-01

**Authors:** Samrat Shrestha, Sabin K. Ghimire, Mecklina Shrestha, Suresh Maharjan, Kiran Bishwakarma

**Affiliations:** a National Academy of Medical Sciences, NAMS, Bir Hospital, Department of General Surgery, Kathmandu, Province-3, Nepal; b College of Medical Sciences (CoMS), Department of Emergency Medicine, Bharatpur, Nepal, Province No. 3, Kathmandu, Nepal

**Keywords:** Case report, Hydatid cyst, echinococcus, Intraperitoneal rupture, Laparotomy, Daughter cyst

## Abstract

**Introduction and importance:**

Hydatid disease, caused by *Echinococcus* larvae, leads to cystic echinococcosis, primarily affecting the liver and lungs. It is common in endemic regions like Argentina and East Africa, with incidences of over 50 cases per 100,000 people. A rare and life-threatening complication is the intraperitoneal rupture of a hydatid cyst.

**Case presentation:**

A 38-year-old female presented with epigastric pain followed by sudden-onset abdominal pain and hypotension. Lab results showed elevated white blood cell count with a high eosinophil. Ultrasonography (USG) and Computed Tomography (CT) scans showed a large, ruptured hydatid cyst of the liver. Emergency laparotomy revealed a ruptured hydatid cyst at the anterosuperior aspect of the liver. The intracystic collection was aspirated, and the cyst cavity and peritoneal cavity were washed with 20 % warm hypertonic saline. Postoperatively, albendazole treatment was started. Follow-up evaluations at 1, 3, and 6 months showed no evidence of recurrence on radiological scans.

**Clinical discussion:**

Intraperitoneal rupture of hydatid cysts is a rare yet life-threatening complication, accounting for 1–16 % of cases. USG and Contrast-Enhanced CT (CECT) of the abdomen are the mainstays for diagnosis, and magnetic resonance cholangiopancreatography (MRCP) helps detect cystobiliary communication. Treatment includes stabilization, surgical cyst evacuation, and scolicidal irrigation. Postoperative albendazole therapy, along with regular follow-up, is essential to prevent recurrence.

**Conclusion:**

Spontaneous rupture of hepatic hydatid cyst with cystobiliary communication is a rare but critical condition, especially in endemic areas, presenting with acute abdomen and shock. Lifesaving emergency laparotomy followed by comprehensive postoperative care is vital for preventing complications and recurrence.

## Highlights

• Intraperitoneal rupture of a hydatid cyst is a potentially fatal and calls for immediate medical attention and/or surgery.
• Mild allergic reactions are common, but anaphylaxis may occur, requiring urgent intervention.
• Surgery aims to eliminate all cyst contents, manage biliary communications, reduce recurrences.
• Postoperative albendazole 10-15 mg/kg in two divided doses is recommended to prevent recurrence.
• Long-term monitoring is crucial since recurring disease can appear years after the initial treatment.

## 1 Introduction

Hydatid disease is a cyclozoonosis transmitted by the fecal-oral route and caused by the larva of a cestode belonging to the genus *Echinococcus*, mainly *E. granulosus*, causing cystic echinococcosis (hydatid cyst) and E. multilocularis, causing alveolar echinococcosis. Overall, hydatid cysts consist of 95 % of all hydatid diseases, most frequently involving the liver (50–77 %) and lungs (15–47 %) [1]. In endemic zones like Argentina, Peru, East Africa, Central Asia, and China, the annual incidence of cystic echinococcosis is >50 per 100,000 person-year [2]. Intraperitoneal rupture of a hydatid cyst is a rare but life-threatening complication requiring emergent medical and/or surgical management [1]. We are reporting a similar case of a 38-year-old female with sudden onset abdominal pain with hypotension. The clinical diagnosis of an intraperitoneal ruptured hydatid cyst of the liver was confirmed on ultrasonography (USG) and contrast-enhanced computed tomography (CECT) of the abdomen. This case report has been reported according to the revised SCARE guidelines, 2023 [3].

## 2 Case presentation

**Fig. 1:**
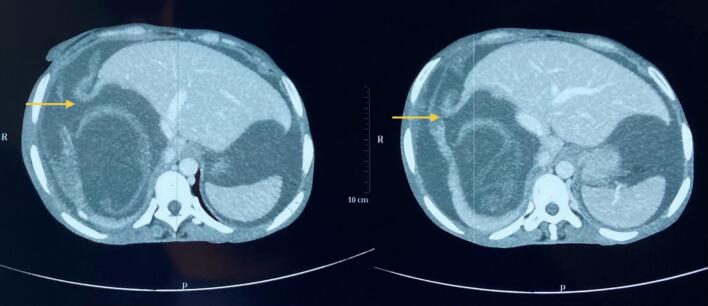
CECT Abdomen: Axial section showing thick-walled cystic lesion in the right lobe of the liver with a defect in the anterosuperior aspect of its wall (yellow arrow). CECT: Contrast Enhanced Computed Tomography. (For interpretation of the references to colour in this figure legend, the reader is referred to the web version of this article.)

**Fig. 2:**
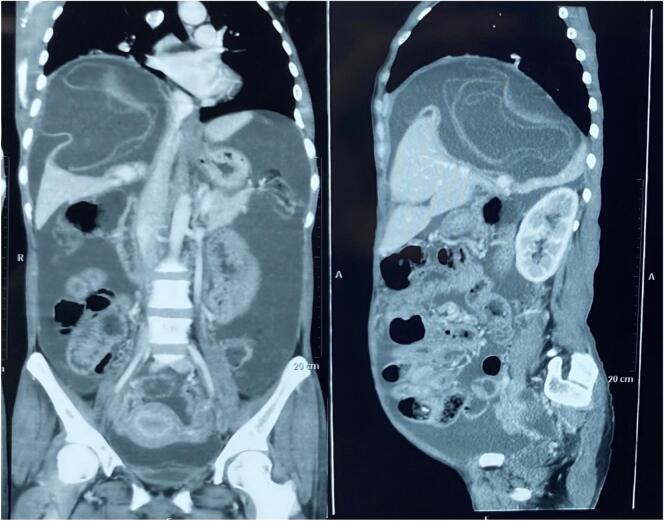
CECT Abdomen: Coronal (A) and Sagittal (B) section showing ruptured hydatid cyst of the liver with collection in the peritoneal cavity. CECT: Contrast Enhanced Computed Tomography.

**Fig. 3:**
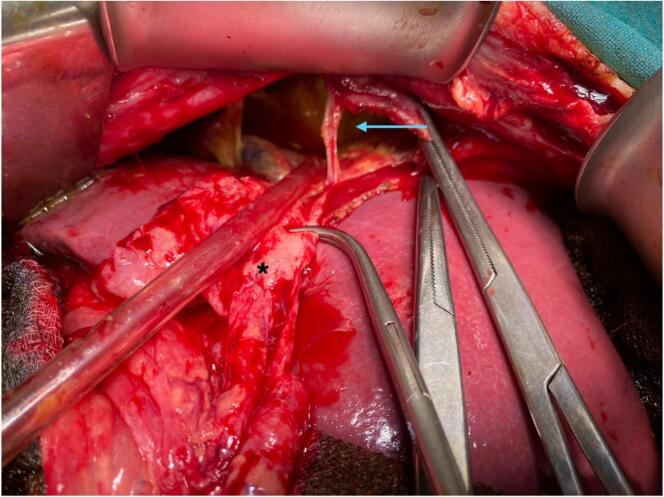
Omental migration (asterisk) into ruptured hydatid cyst cavity (blue arrow). (For interpretation of the references to colour in this figure legend, the reader is referred to the web version of this article.)

**Fig. 4:**
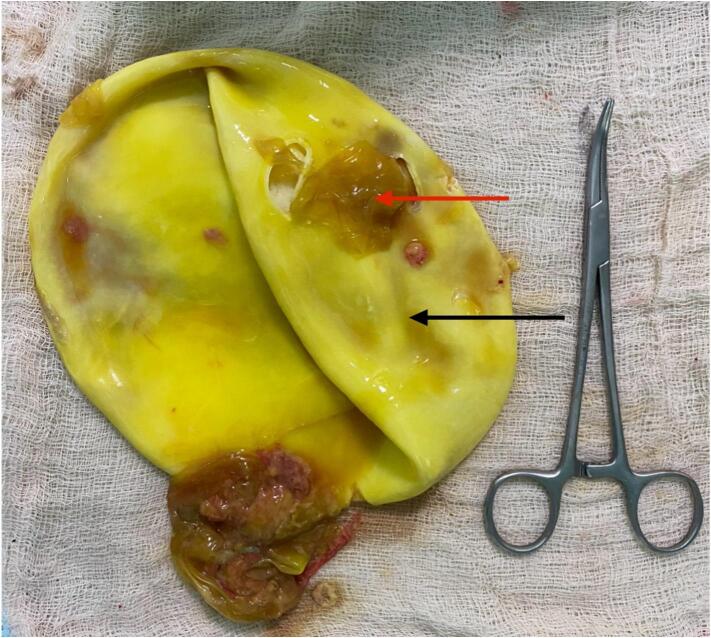
Laminated membrane (black arrow) of hydatid cyst with ruptured daughter cyst (red arrow). (For interpretation of the references to colour in this figure legend, the reader is referred to the web version of this article.)

**Fig. 5:**
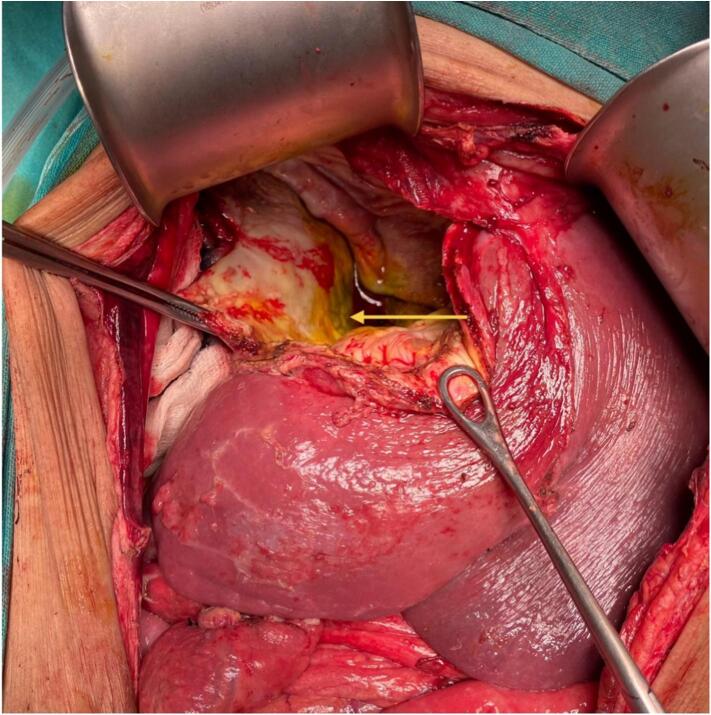
Intraoperative picture of ruptured hydatid cyst located at segments VII and VIII of the liver with cystobiliary communication marked by a bile-stained cystic cavity (yellow arrow). (For interpretation of the references to colour in this figure legend, the reader is referred to the web version of this article.)

A 38-year-old female presented to the emergency department of our hospital with dull, aching epigastric pain for 3 weeks and later sudden-onset generalized abdominal pain, which started 2 days before hospital admission. She had a history of livestock (sheep) grazing. She had no history of fever or allergic reaction. The patient was hypotensive (blood pressure 80/40 mmHg). On abdominal examination, the patient had epigastric fullness with tenderness mainly at the epigastric and right upper quadrant. Fluid resuscitation was initiated to stabilize the patient. The patient's laboratory parameters were hemoglobin: 10.9 g/dl, total leukocyte count: 15050/cumm, neutrophil: 65 %, lymphocyte: 10 %, and eosinophil: 30 %. The patient's liver function test was total bilirubin: 0.7 mg/dl; direct bilirubin: 0.3 mg/dl; alanine aminotransferase (ALT): 26 IU/L; aspartate aminotransferase (AST): 26 IU/L; alkaline phosphatase: 155 IU/L; total protein: 4.3 g/dl and albumin: 2 g/dl. Other laboratory parameters were within normal limits. USG of the abdomen revealed a large cystosolid lesion in the liver with internal vascularity and moderate free fluid collection in the peritoneal cavity, suggesting a hydatid cyst of the liver. CECT showed a well-defined, thick-walled cystic lesion (13cm x12cm x11cm) in the right lobe of the liver involving segments VII and VIII and multiple daughter cysts within the cystic cavity with a defect of 11.5 mm in the anterosuperior aspect of its wall with gross ascites suggestive of a ruptured hydatid cyst (Fig. 1, Fig. 2). CECT chest was done to rule out the hydatid cyst of the lung. The echinococcal ELISA IgG antibody was positive. The diagnosis of intraperitoneal rupture of hydatic cyst of the liver was made, and an emergency exploratory laparotomy was planned. The patient expressed significant concern upon diagnosis of a ruptured hydatid cyst, fearing surgery. However, she appreciated the clear communication from the surgical team regarding the risks and treatment options. Informed consent was obtained from the patient after a detailed discussion about the risks, benefits, and alternatives to surgery for the ruptured hydatid cyst. The patient verbally agreed and signed the consent form for the planned surgical procedure. During laparotomy, approximately 5 l of bilious collection containing cyst material were aspirated from the peritoneal cavity. There was omental migration into a ruptured hydatid cyst cavity (Fig. 3) with a defect present at the anterosuperior aspect of the liver involving segments VII and VIII and a cystic cavity consisting of a laminated membrane (Fig. 4) containing the daughter cyst and intracystic bilious collection. The intracystic collection was aspirated, the cyst component was completely evacuated, and the cyst cavity and peritoneal cavity were washed with 20 % warm hypertonic saline. Minor cystobiliary communications (Fig. 5) were noted at 3 points, which were repaired using absorbable polyglactin 910 sutures. 2 abdominal drains were kept, one at the cystic cavity and the other at the pelvic cavity, and omentopexy was done. The patient was then shifted to the intensive care unit for further monitoring. Postoperative albendazole at 15 mg/kg was started in 2 divided doses (400 mg BD). The abdominal drains were removed after the 10th POD, and the patient was discharged at the 14th POD. Albendazole 400 mg was prescribed on discharge for 3 months. After surgery, her recovery was gradual, though she continued to experience concerns about recurrence, which were addressed during follow-up visits. On 1-month, 3-month, and 6-month follow-ups, the patient showed no sign of recurrence on radiological evaluation.

## 3 Discussion

Liver echinococcosis accounts for 60–75 % of cases, with 80 % located in the right lobe [4]. The majority of them are asymptomatic and are diagnosed incidentally during routine checkups. Symptomatic cysts either present with right upper abdominal pain, abdominal distension, and discomfort or present with complications such as secondary infection, rupture, or pressure effect on the adjacent organ or tissue [5]. Three types of rupture have been described to date: contained, communicating, and direct rupture [6]. Contained rupture occurs when a ruptured cyst remains contained by the adjacent hepatic parenchyma [7]. Communicating rupture occurs when the bile ducts are eroded by the expanding cyst, leading to communication with the cyst cavity and leakage of bile into the cyst. It is reported in about 26–34 % of cases, with most involving the right lobe [8]. Intraperitoneal rupture is an uncommon yet critical complication that poses significant diagnostic and therapeutic challenges [1]. It is found in 1–16 % of the reported cases [7]. It may result from trauma or may occur spontaneously from increased pressure of the cystic fluid (Spontaneous rupture in our case). The predisposing factors for rupture include young age, cyst diameter > 10 cm, presence of cysto-biliary communication (CBC), and superficial cyst location [9,10]. Patients with intraperitoneal rupture often exhibit acute abdominal pain, abdominal distension, nausea, vomiting, and fever. Signs of peritonitis, including hypotension, tenderness, and guarding, may be present [11]. Obstructive jaundice, biliary colic, or features of cholangitis suggest major CBC (5–17 % of cases). A minor CBC (10–37 % of cases) is usually asymptomatic and is diagnosed intraoperatively or postoperatively by an external biliary fistula [[12], [13], [14]]. Around 16.7 %–25 % of patients with intraperitoneal rupture have mild allergic reactions, like urticaria or rashes, while 1 %–12.5 % experience severe reactions, including peripheral edema, syncope, and anaphylaxis requiring urgent intervention [1]. USG of the abdomen and pelvis can detect cysts as well as suspect rupture by showing a floating membrane with intraperitoneal fluid. CECT may be used in hemodynamically stable patients as it is very effective in identifying cyst rupture, peritoneal free fluid, and the presence of daughter cysts. The sensitivity of USG and CT in identifying cyst rupture is 85 % and 100 %, respectively [15]. For detecting CBC, magnetic resonance cholangiopancreatography (MRCP) has proven to be a reliable modality, allowing detailed visualization of biliary structures and highlighting areas where the cyst may have eroded into the biliary ducts. Elevated alkaline phosphatase levels and bilirubin levels can further support the diagnosis of CBC [16]. Management of intraperitoneal rupture involves immediate medical therapy with benzimidazole; antihistaminic to prevent anaphylaxis; intravenous fluids, corticosteroids, and oxygen to stabilize hemodynamics followed by surgery. The goal of surgery is to remove all the cyst contents, control biliary leakage, minimize morbidity and mortality risk, and prevent secondary echinococcosis and recurrences [17]. Both laparoscopic and open surgical approaches are preferred in hemodynamically stable patients. [18] Open surgical approach is more suitable for hemodynamically unstable patients. A more conservative surgery like partial pericystectomy and deroofing is preferred to radical pericystectomy. During emergency operations, a complete evaluation of the abdominal cavity should be performed. Aspiration of all cyst contents followed by irrigation of the abdominal and cyst cavities with scolicidal agents should be done to minimize secondary peritoneal hydatidosis and anaphylactic reaction. The common scolicidal agents in use are hypertonic saline (3–30 %), silver cetrimide + chlorhexidine (1.5 % + 0.15 %), and povidone‑iodine (10 %) [18]. Minor CBC in cyst cavities (<5 mm diameter) can be treated by suturing it on the healthy tissue after removal of the capsule of the hydatid cyst along with drainage. Major CBD (>5 mm diameter or communication with major bile ducts) requires common bile duct exploration and T-tube drainage. In patients with postoperative bile leakage, early ERCP (sphincterotomy ± stenting) is an effective diagnostic and therapeutic option [19]. Mortality and morbidity range from 0 to 23.5 % and 20 % to 35.3 %, respectively [20]. Antiparasitic therapy with albendazole 10–15 mg/kg in two divided doses is recommended postoperatively to prevent recurrence. Although there is controversial data in the literature, it is suggested that anthelminthic therapy should be continued for 1 to 12 months after the operation [18]. Long-term follow-up is essential, as recurrent disease can develop even years after initial treatment in 6.7 % to 28.6 % of cases [20]. Echinococcus IgG ELISA or indirect hemagglutination (IHA) can be performed for postoperative surveillance. The patients are evaluated every three months for the first year, every six months for the second year, and annually thereafter [18]. Various studies highlight the critical nature of intraperitoneal rupture of hydatid cysts and the need for prompt diagnosis and surgical intervention. Our case aligns well with the series from Mejri et al. and Dirican et al., where acute abdominal pain and shock are prominent features [7,9]. Our case highlights the life-threatening nature of intraperitoneal rupture in a patient with cystobiliary communication, emphasizing the importance of emergency laparotomy, scolicidal lavage, and postoperative care with albendazole. The detailed exploration of cystobiliary communication during surgery adds value to the literature by providing insights into managing this rare and potentially dangerous complication.

## 4 Conclusion

The spontaneous rupture of a hydatid cyst of the liver into the peritoneum with cystobiliary communication represents a rare but life-threatening complication requiring urgent intervention. A high index of suspicion should be made in a patient residing in the endemic area who presents with an acute abdomen. Our patient, who presented with shock and peritonitis, underwent a life-saving emergency exploratory laparotomy. Prompt surgical exploration facilitated the evacuation of the cyst contents, management of biliary contamination and communication, and thorough peritoneal lavage, which are critical steps to minimize the risk of persistent infection, secondary hydatidosis, and sepsis. In endemic areas, increasing awareness among healthcare providers about hydatid disease and its complications can lead to earlier detection and more successful management. This case highlights the importance of early recognition and decisive surgical management in improving outcomes for patients with this life-threatening presentation. Additionally, postoperative care, including close monitoring for residual biliary fistula and antiparasitic therapy, remains essential to prevent recurrence and ensure complete recovery.

## CRediT authorship contribution statement

1. Constructing hypothesis for the manuscript- Samrat Shrestha, Sabin K. Ghimire
2. Planning methodology to reach the conclusion: Samrat Shrestha, Sabin K. Ghimire, Mecklina Shrestha, Suresh Maharjan, Kiran Bishwakarma
3. Organizing and supervising the course of the article and taking responsibility: Samrat Shrestha.
4. Patient follow-up and reporting – Mecklina Shrestha, Sabin K. Ghimire, Suresh Maharjan, Kiran Bishwakarma.
5. Logical interpretation and presentation of the results- Samrat Shrestha, Sabin K. Ghimire, Mecklina Shrestha, Suresh Maharjan, Kiran Bishwakarma.
6. Construction of the whole or body of the manuscript- Samrat Shrestha, Sabin K. Ghimire, Mecklina Shrestha, Suresh Maharjan, Kiran Bishwakarma.
7. Reviewing the article before submission not only for spelling and grammar but also for its intellectual content- Samrat Shrestha, Sabin K. Ghimire, Mecklina Shrestha, Suresh Maharjan, Kiran Bishwakarma.

## Consent

Written informed consent was taken from the patient who participated in this study for publication of this case report and accompanying images.

## Ethical approval

The IRB at our institution has waived ethical approval for case reports.

## Guarantor

The guarantor is Samrat Shrestha.

## Sources of funding

There are no sources of funding for this case study to declare.

## Declaration of competing interest

The authors have no conflict of interest to declare.
